# Survival and Replication of Zika Virus in Diapause Eggs of *Aedes Albopictus* From Beijing, China

**DOI:** 10.3389/fmicb.2022.924334

**Published:** 2022-07-07

**Authors:** Qianghui Zhang, Yuting Jiang, Chaojie Li, Jian Gao, Teng Zhao, Hengduan Zhang, Chunxiao Li, Dan Xing, Yande Dong, Tongyan Zhao, Xiaoxia Guo

**Affiliations:** Department of Vector Biology and Control, State Key Laboratory of Pathogen and Biosecurity, Beijing Key Laboratory, Institute of Microbiology and Epidemiology, Beijing, China

**Keywords:** ZIKV, *Aedes albopictus*, photoperiod, diapause, vertical transmission

## Abstract

Zika virus (ZIKV) has emerged as a globally important arbovirus. The virus is primarily transmitted to humans through the bite of an infective *Aedes albopictus* in temperate area. Vertical transmission of ZIKV by *Ae. albopictus* is determined and has been suggested to be a means by which the virus could persist in nature. *Ae. albopictus* undergoes a well-characterized photoperiodic diapause. Viruses are harbored by overwintering mosquitoes in diapause that contributes to the resurgence of vertebrate diseases in the following spring, yet little is known about the impact of diapause on the regulation of viral replication and survival. The purpose of this study is to determine that *Ae. albopictus* in Beijing are highly susceptible to ZIKV (92.3%), and viable virus is passed to their organs of progeny *via* vertical transmission. Moreover, diapause eggs (diapause incidence 97.8%) had significantly lower minimum infection rates and filial infection rates of the first gonotrophic cycle than those of the second gonotrophic cycle in the short-day photoperiod group. Regarding the development of diapause eggs, the minimum infection rates and ZIKV RNA copy number increased significantly, suggesting that virus RNA replication occurred in the diapause eggs. Meanwhile, eggs from the ZIKV-infected mosquitoes had a significantly lower hatching rate compared with uninfected mosquitoes, implying an intriguing interaction between diapause eggs and virus. The findings here suggest that vertical transmission of ZIKV from diapause eggs to progeny may have a critical epidemiological role in the dissemination and maintenance of ZIKV circulating in the vector.

## Introduction

Zika virus (ZIKV) is an emerging mosquito-borne virus belonging to the genus *Flavivirus* of the Family Flaviviridae (Kuno et al., 1998). It was first isolated from a febrile sentinel rhesus monkey in Uganda in 1947 (Dick and Haddow, 1952). Following the 2016 outbreak in the Americas, the World Health Organization declared that the ZIKV outbreak constituted a Public Health Emergency of International Concern (Musso and Gubler, 2016). With regard to how ZIKV was able to be transmitted from Africa to Asia and then to the Americas, one possible explanation is that its vector mosquitoes *Aedes albopictus* are capable of vertically transmitting ZIKV, which may have contributed to its rapid global range expansion (Venceslau et al., 2020). In addition, travel by infected humans is also a known pathway for transporting arbovirus to new geographic locations with the development of globalization and urbanization (Thomas et al., 2014).

The Asian tiger mosquito, *Ae. albopictus*, is a highly invasive mosquito that has spread from its native Southeast Asia to North America, South America, Europe, and Africa over the last 30 years (Lounibos, 2002; Benedict et al., 2007). It is competent to transmit at least 22 arboviruses in addition to dengue virus, chikungunya virus, and ZIKV that cause human illness (Gratz, 2004; Delatte et al., 2008; Paupy et al., 2009). *Ae. albopictus* is widely distributed in both subtropical and temperate zones (Bonizzoni et al., 2013). Diapause is an alternative life history strategy of many mosquito species, especially *Ae. albopictus*. Diapause eggs in *Ae. albopictus* are both more resistant to cold temperatures and desiccation-tolerant than non-diapause eggs due to accumulating extra nutrients and lipids for energy conservation and utilization, which facilitate *Ae. albopictus* to survive during the harsh conditions (Urbanski et al., 2010). The capacity of this mosquito to colonize and establish in new areas (including temperate area) is enhanced by its ability to produce diapausing eggs that survive in relatively cold winters (de Carvalho et al., 1981). Photoperiod and temperature were the primary influential factor for diapause (Urbanski et al., 2012). Xia et al. reported that Guangzhou population of *Ae. albopictus* was induced diapause by a short-day (SD) photoperiod in the laboratory, and diapause eggs in the field were the main form for overwintering and began to hatch in large quantities in March (Xia et al., 2018). Diapause in *Ae. albopictus* offers a mechanism for bridging unfavorable seasons in both temperate and subtropical environments and serves to synchronize development within populations, thus directly affecting disease transmission cycles (Denlinger and Armbruster, 2014).

Previous experimental studies have adequately assessed that diapausing stages are critical for harboring the viruses during winter and subsequently reintroducing them as disease agents in the following summer for diseases such as West Nile virus and La Crosse virus (Benedict et al., 2007). A study conducted in a city in Brazil provided evidence of ZIKV detection in field-collected *Aedes aegypti* eggs during the emergence of ZIKV in 2015–2016. The result indicated the existence of natural vertical transmission may contribute to ZIKV maintenance in nature during epidemic periods (da Costa et al., 2018). Imported viral diseases increasing year by year are becoming a serious public health threat in China. There were 24 imported cases of Zika in China, including Beijing and Guangdong (Wang et al., 2018b). Vertical transmission of ZIKV in *Ae. albopictus* was also confirmed recently (Lai et al., 2020). However, the importance of ZIKV infection specifically in *Ae. albopictus* diapause eggs in the virus maintenance during unfavorable environmental conditions has not been investigated yet. This study revealed the possible role of infected diapause eggs of *Ae. albopictus* in Beijing in the maintenance of the ZIKV against adverse climatic conditions and ZIKV vertical transmission occurred through *Ae. albopictus* diapause eggs to offspring.

## Materials and Methods

### Colonization of Mosquitoes

The *Ae. albopictus* strain used in this experiment was collected as larvae from different sites in Beijing (N39°51′, E116°17′) in 2019. The laboratory colony was maintained for five generations under insectary conditions of 26 ± 1°C, 70–80% relative humidity (RH), and a non-diapause- inducing long-day photoperiod (LD) of 16 h:8 h light:dark (16L:8D) cycles. Adults were held in rearing cages (25 cm × 25 cm × 25 cm cage covered with nylon mesh) and provided with 8% sucrose solution.

### ZIKV and Cells

The ZIKV SZ01 strain (GenBank no. KU866423), provided by the Microbial Culture Collection Center of the Beijing Institute of Microbiology and Epidemiology, was isolated from a patient who returned from Samoa to China in 2016 (Deng et al., 2016). Virus was harvested and separated into 1 ml aliquots stored at −80°C, which had been passaged five times through C6/36 cells. Viral titer of infectious blood meal was 7.1 × 10^7^ PFU/ml *via* plaque-forming assay in baby hamster kidney cells.

### Oral Infection

Prior to offering them an infectious meal, 5–7-day-old female mosquitoes were deprived of sucrose solution for 16 h. The infectious blood meal was a mixture of fresh virus suspension and mouse blood at a ratio of 1:1. While feeding, the viral blood meal was constantly warmed to 37°C using a Hemotek membrane feeding system. After 30 min exposure to the blood meal, mosquitoes were CO_2_ anesthetized and only fully engorge females (over 500 individuals per cage) were transferred immediately to cages. The LD females were daily given 10% sucrose in a chamber at 28°C and 80% RH under LD photoperiods. In contrast, the females under the diapause-inducing SD photoperiod were kept in the same environment. Besides, uninfected control cages under LD and SD conditions were fed with the uninfected blood meal consisting of 1:1 mouse blood and Dulbecco's modified Eagle's medium (Gibco?; Invitrogen, Beijing, China), which were treated in parallel to the infectious blood-fed cages.

### Vector Competence of the *Ae. albopictus* to ZIKV

To demonstrate vector competence, about 24 mosquitoes from the LD group were selected on 0, 4, 7, 10, and 14 days post-infection (dpi). Salivary glands and ovaries were tested to assess the dissemination rate (DR). The saliva of infected females was processed to assess the transmission rate (TR). The salivary glands, midguts, and ovaries were carefully dissected and washed three times in phosphate-buffered saline. Saliva was collected from individual mosquito at the same time (Dubrulle et al., 2009). Every tissue was placed in 1.5 ml tubes containing 100 μl Trizol, stored at −80°C.

### Oviposition

**Figure 1:**
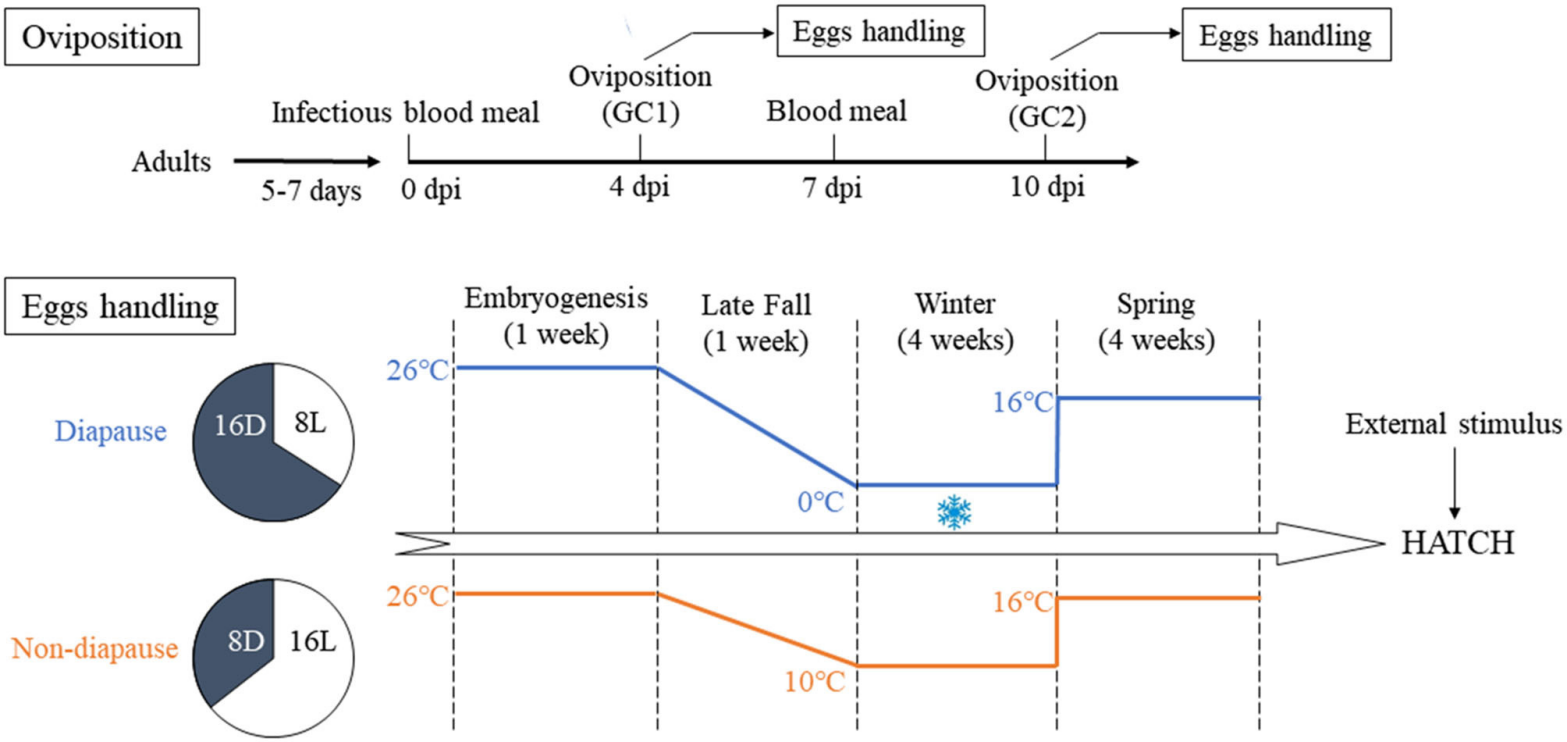
Illustration of the experimental design in the context of oviposition and eggs handling. GC1, first gonotrophic cycle; GC2, second gonotrophic cycle.

An overview of the experimental design is presented in Figure 1. Briefly, four cohorts (biological replicates) of the *Ae. albopictus* were reared under LD conditions described above. Meanwhile, four cohorts of the newly hatched three instar larvae were established under a diapause-inducing SD photoperiod of a light:dark cycle of 8 h:16 h (8L:16D) until the emergence. After blood meal, filter paper used as an oviposition substrate in small containers of deionized water was placed in cages on 4 dpi. The eggs on the filter paper from the first gonotrophic cycle (GC1) were collected and air-dried. To allow complete embryonation, the eggs were kept for 7 days under laboratory conditions (Hanson and Craig, 1994). The females only ingested mouse blood after the first oviposition. On 10 dpi, the eggs from the second gonotrophic cycle (GC2) were collected in the same way as mentioned above prior to preserving the eggs. For each group, eggs collected from biological replicate egg paper were divided into samples of about 500 eggs each and respectively assigned to SD photoperiod and LD photoperiod groups.

### Diapause Induction

The eggs from GC1 and GC2 of the SD diapause-inducing group were held in an illumination incubator (MGC-300B, Shanghai Yiheng Co., Ltd., Shanghai, China) and subjected to SD conditions and 90% RH, while the temperature was decreased from 26 to 0°C during 1 week like the environment of late fall. Then, the eggs were sustained at 0°C for 4 weeks to simulate the winter environment of Beijing City. The eggs of LD non-diapause group were maintained at 10°C. The time span of LD group was the same as SD group. After that, all eggs in the SD and LD groups were still kept and developed for 4 weeks by establishing a simulated spring climate in which the temperature was raised to 16°C and the photoperiod was changed to LD conditions. Eggs of uninfected group were maintained under LD and SD conditions in equal way.

To terminate diapause, the eggs of duplicated cohorts were hatched by submerging in food slurry (Poelchau et al., 2014). After counting hatched larvae, unhatched eggs were air-dried and returned to 16°C under LD conditions. Finally, unhatched eggs were re-stimulated to hatch 7 days later in order to record the number of embryonated unhatched eggs. Hatching rates were calculated as the percentage of hatched eggs to the total eggs. The remaining embryonated unhatched eggs were bleached (Trpiš, 1970). Diapause incidence was calculated as the total number of embryonated eggs divided by the number of embryonated unhatched eggs. Diapause eggs were identified using an optical microscopy (MXB-2500REX; Hirox, Tokyo, Japan) at 200× magnification.

### Mosquito Eggs Collection

To make out the level of infectivity in the eggs, we calculated the minimum infectivity rate (MIR) to identify vertical transmission of ZIKV. MIR is the ratio of the number of positive pools to the total number of individuals (eggs) tested. Each positive pool indicated that one or more of the eggs in the pool were infected with ZIKV. On 3, 7, 11, 21, 40, and 70 days post-oviposition (dpov), we sampled about 24 pools (30 unbroken eggs per pool) of the above eggs for RNA extraction and covered the GC1 and GC2 from LD and SD conditions. The time points were chosen for the different developmental stage. The 3 dpov period represented clear morphological landmarks in embryonic development, with the developmental landmarks of germ band retraction and dorsal closure; and the 7 dpov period comprised the end of embryonic development with cleared embryos, which were competent to hatch. On 11 dpov, developmental arrest was firmly established in diapause embryos (early diapause). Eggs were in mid-diapause on 21 dpov and late diapause or post diapause quiescence on 40 dpov (Poelchau et al., 2013).

Moderate eggs were simultaneously retained to quantify the titers for different GC to confirm the existence of ZIKV. Once the samples were positive, these eggs were grouped and macerated in RPMI 1640 (Gibco?; Invitrogen) with 1% penicillin/streptomycin (10,000 U/ml) and 2% fetal bovine serine, and were cultivated in C6/36 cells to isolate the virus. Observation of ZIKV growth and the cytopathic effects was monitored 5 days later as described above (Contreras and Arumugaswami, 2016). The ZIKV-free eggs were treated and inoculated into cell culture at the same time as negative control groups. In addition, the ZIKV RNA was detected by RT-qPCR.

### Filial Infection Rate (FIR)

To further evaluate the existence of vertical transmission of ZIKV, FIR of ZIKV was detected to calculate the FIR (the proportion of infected larvae or adults in the total tested individuals). We collected some eggs from all groups after the above treatment. After 70-day-old eggs were hatched, 24 pools of the 3–4 instar larvae (five larvae per pool) and 24 pools of individual adult females (five adults per pool) were determined for ZIKV by qPCR. We also dissected the salivary glands, midguts, and ovaries of freshly emerged F1 female adults for detection. The saliva was obtained using before methods. In addition, other offspring mosquitoes were transferred to a cage and bit 10 neonatal mice. After the observation of symptoms, neonatal mouse brains were extracted for RNA. To confirm the viability of infectious ZIKV in the progeny, the samples consisting of ZIKV-positive adult offspring were used to isolate the virus by inoculating C6/36 cells.

### Quantification of ZIKV

The total RNA was extracted using the QIAamp Viral RNA Mini Kit (QIAGEN, Hilden, Germany) following the protocol of the manufacturer after tissues were ground by a freeze-grinding machine (60 MHz, 2 min) (HODER Beijing N. 9548R). One-step nucleic acid detection kit (DaAn Gene, Guangzhou, China) was used to detect the RNA genome, which was determined by absolute RT-qPCR on a 7500 Real-time Quantitative PCR System (Applied Biosystems, Foster City, USA) under the following program, namely, 1 cycle at 50°C for 15 min, 95°C for 15 min; 40 cycles at 94°C for 15 s, and 55°C for 45 s. The number of viral RNA copy was calculated by generating a standard curve as previously described (Li et al., 2017). Viral RNA copy number was expressed as log_10_ RNA copies per μl.

### Statistical Analysis

All statistical analyses were conducted using R version 3.6.1 and SAS version 9.3 (Cary, NC, USA). Chi-square and Fisher's exact tests were used to compare the rates. Multiple comparisons of the rates were performed with R using rcompanion packages. The ZIKV RNA copy number was log-transformed and then compared using two-way ANOVA *post-hoc* Tukey tests. Significance of *p*-value was corrected by Bonferroni adjustments, and *p* < 0.05 was considered significant.

## Results

### Vector Competence of Mosquitoes to ZIKV Through Oral Infection

**Figure 2:**
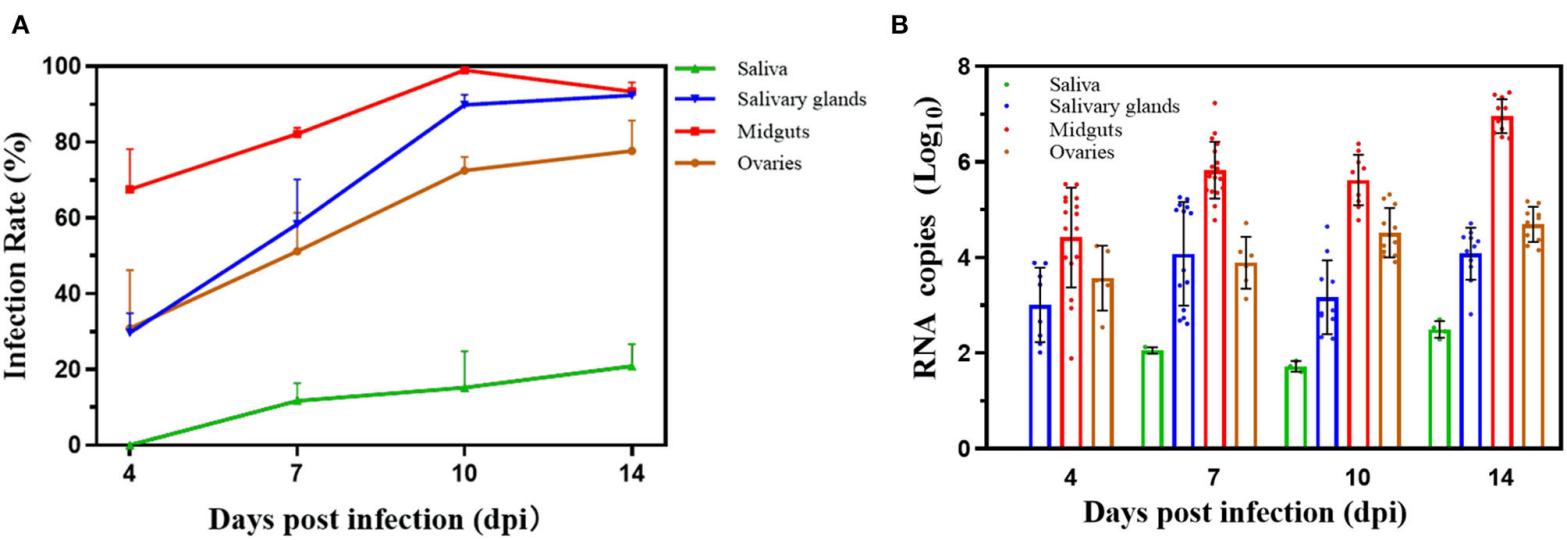
Infection rates and virus reproduction for Zika virus in Beijing strain. **(A)** Vector competence of Zika virus in Beijing strain. The saliva, salivary glands, midguts, and ovaries were dissected on 0, 4, 7, 10, and 14 days post infection, and Zika virus was detected by reverse transcription quantitative real-time polymerase chain reaction. The results are expressed as mean ± SD. **(B)** Zika virus RNA copy number in infected saliva, salivary glands, midguts, and ovaries. Each point represents one positive tissue. *X*-axis represents days post infection (dpi), and *Y-*axis represents RNA copy number. Error bars indicate mean ± SD.

The infection rates were calculated by detecting infection status of midguts, saliva, and salivary glands or ovaries for ZIKV to evaluate vector competence. ~288 female mosquitoes (96 mosquitoes per duplicated trial) were infected by ZIKV, and the selected 30 females with full abdomen were ZIKV positive by qPCR after first infectious blood-fed (0 dpi). The results suggested that all midguts were infected with ZIKV because of the undigested viral blood meal on 0 dpi, while no virus was detected in other tissues (Figure 2A). The TR and DR continually increased with time. However, the difference in the TR was not significant on 7, 10, and 14 dpi (Fisher's exact test, *p* > 0.05). The IR of midguts, which rose from 4 dpi and rapidly increased to peak near 100% on 10 dpi and then declined on 14 dpi, was not accumulated. But the IR of 10 dpi was significantly higher than those of either 4 or 7 dpi (*p* < 0.01, *p* < 0.05). ZIKV could also be measured in the saliva glands and ovaries, which were significantly lower DR of 4 dpi than those of 10 dpi (*p* < 0.05) and 14 dpi (*p* < 0.01). There was relatively lower infection rate of the saliva glands than rates of other tissues, though the rates on 10 and 14 dpi were higher compared with the rates on 7 dpi (*p* < 0.05, *p* < 0.05).

The RNA copy number in the saliva, salivary glands, midguts, and ovaries had significant differences, respectively. Virus was detected in the midguts, salivary glands, and ovaries from 4 dpi, except the saliva (Figure 2B). The RNA copy number in the saliva on 10 dpi was lower than RNA copy number on 14 dpi (*p* > 0.05). The amount of RNA copy in saliva glands increased from 4 dpi (3.0 ± 0.78) to 14 dpi (4.13 ± 0.54) with slight fluctuations (*p* > 0.05). But the RNA copy number of midguts rapidly raised to 6.91 ± 0.36 log_10_ RNA copies/μl on 14 dpi, which was significantly higher than those on 4 dpi (4.42 ± 1.01, *p* < 0.01), 7 dpi (5.83 ± 0.58, *p* < 0.01), and 10 dpi (5.59 ± 0.51, *p* < 0.01). For ovaries, the trend of the RNA copy number was slowly increasing to 4.74 ± 0.36 log_10_ RNA copies/μl on 14 dpi, significantly higher than that before (*p* < 0.05).

### MIR of Eggs

**Figure 3:**
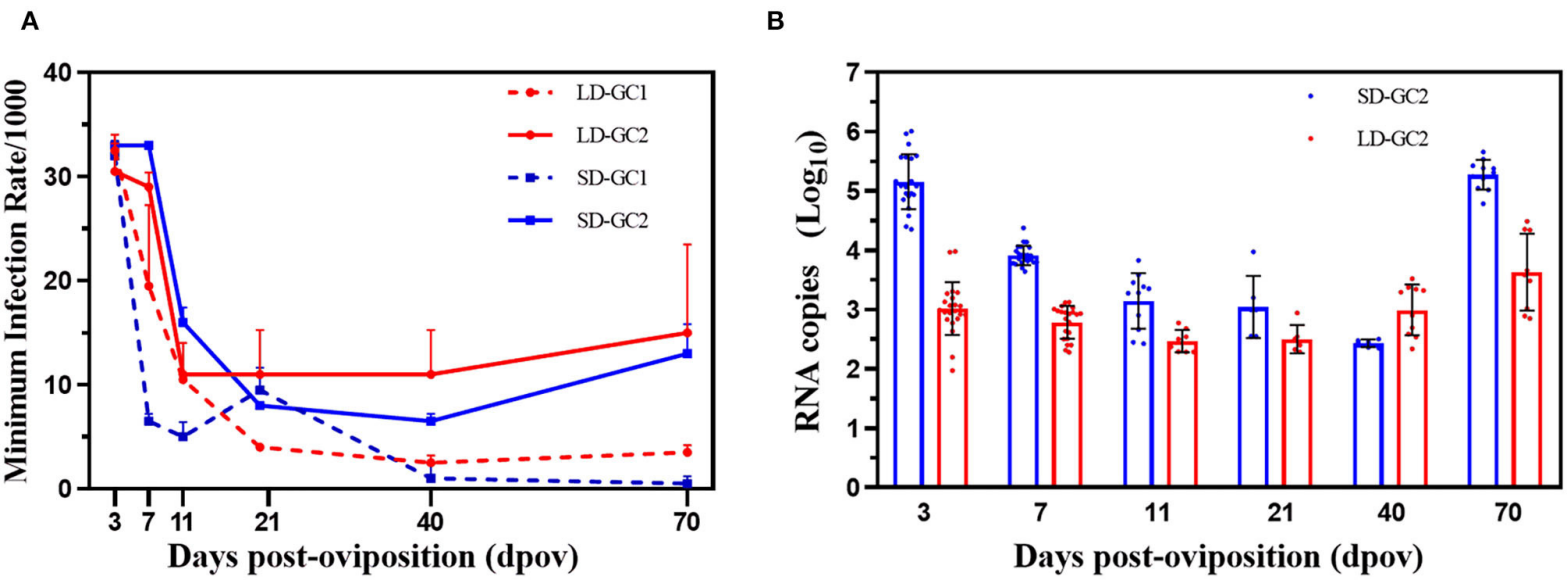
Minimum infection rates and RNA copy number of Zika virus in *Aedes albopictus* mosquitoes on 3, 7, 11, 21, 40, and 70 days post-oviposition. **(A)** Minimum infection rates/1,000 is the ratio of the number of positive pools to 1,000 eggs tested. Red dotted lines indicate the MIRs in the first gonotrophic cycle under long-day photoperiod. Red solid lines depict the MIRs in the second gonotrophic cycle under long-day photoperiod. Blue dotted lines indicate the MIRs in the first gonotrophic cycle under short-day photoperiod. Blue solid lines depict the MIRs in the second gonotrophic cycle under short-day photoperiod. Error bars indicate SDs. **(B)** Zika virus RNA copy number in egg pools on 3, 7, 11, 21, 40, and 70 days post-oviposition. Each point represents one positive pool. Red dots indicate RNA copy number in the second gonotrophic cycle under long-day photoperiod. Blue dots indicate RNA copy number in the second gonotrophic cycle under short-day photoperiod. Error bars indicate SDs.

Details of pools tested for ZIKV in positive pools are shown in Figure 3A. In GC1, the MIRs declined from peak 1/30.7 on 3 dpov to low 1/400 (Fisher's exact test, *p* < 0.01) under LD conditions, with the high proportion before 7 dpov and stay stable during the next 4 weeks. But there was no significant difference between the MIR on 70 dpov and the MIR on 40 dpov after being maintained at 16°C for 1 month. The trend of the MIR in the LD-GC2 group was similar to that of LD-GC1. The MIRs on 3 and 7 dpov were significantly higher than that on 40 dpov (*p* < 0.05). The MIRs of 40 and 70 dpov did not differ significantly (*p* > 0.05) when the temperature was raised. For SD, the MIRs of SD-GC1 were observed with the highest level of ZIKV infection on 3 dpov, which rapidly decreased to 40 dpov (*p* < 0.05). Although we quantified the relatively high MIR (1/32.3) of GC1 on 3 dpov, no ZIKV was found on 40 dpov in repeated trials until only detected once. When returning to LD conditions and 16°C, no significant difference was observed as time went by. However, a significant difference (*p* < 0.01) of the MIRs in SD-GC2 was detected among 3 dpov (1/30.0), 11 dpov (1/62.5), and 40 dpov (1/144.9). Furthermore, the level of MIR increasingly reached 1/77.5 from 40 dpov and was apparently higher after 1 month under LD conditions and 16°C.

The RNA copy number of GC1 in SD group was detected once on 40 dpov, while no eggs were positive for ZIKV in repeated experiments. Therefore, the RNA copy number of GC2 in SD group is presented in Figure 3B. The RNA copy number of SD group obviously continued to plummet after infection. The value continually decreased from 3 dpov (5.12 ± 0.46) and was apparently higher than RNA copy number on 40 dpov (*F* = 6.12, *p* < 0.05). Through 1-month virus duplicate in eggs, the peak copy number, which occurred on 70 dpov (5.27 ± 0.25), was significantly higher than RNA copy number of 40 dpov (*F* = 9.773, *p* < 0.01). Similarly, the level of RNA copy number in LD-GC2 was a slow decline over time and reached 2.75 ± 0.27 log_10_ RNA copies/μl on 40 dpov. Then, the growth in ZIKV became rapid and the ZIKV RNA copy number reached 3.63 ± 0.64 log_10_ RNA copies/μl on 70 dpov due to the rise in temperature (*F* = 4.438, *p* < 0.05).

However, the mean of RNA copy number had a large variation between SD and LD in GC2. The RNA copy number of SD-GC2 reached 5.12 ± 0.46 log_10_ RNA copies/μl on 3 dpov and was higher than those of LD-GC2 (2.99 ± 0.41) (*F* = 15.60, *p* < 0.01). On 7 dpov, the number of viral RNA copy (3.91 ± 0.16) was higher than that on 40 dpov (*F* = 7.03, *p* < 0.01). No significant difference between SD-GC2 and LD-GC2 was found on 11 and 40 dpov (*p* > 0.05), whereas a significant increase in the RNA copy number of SD-GC2 (5.27 ± 0.25) was apparently observed compared with the value of LD-GC2 when the developmental eggs on 70 dpov were detected for RNA (*F* = 7.80, *p* < 0.01).

The supernatants of ZIKV RNA-positive eggs on 70 dpov were collected after homogenization. The viral titers of LD group and SD group were 3.3 × 10^2^ and 1.25 × 10^3^ PFU/ml by plaque assay using BHK cell line, respectively.

### Hatching Rate and Diapause Incidence

**Figure 4:**
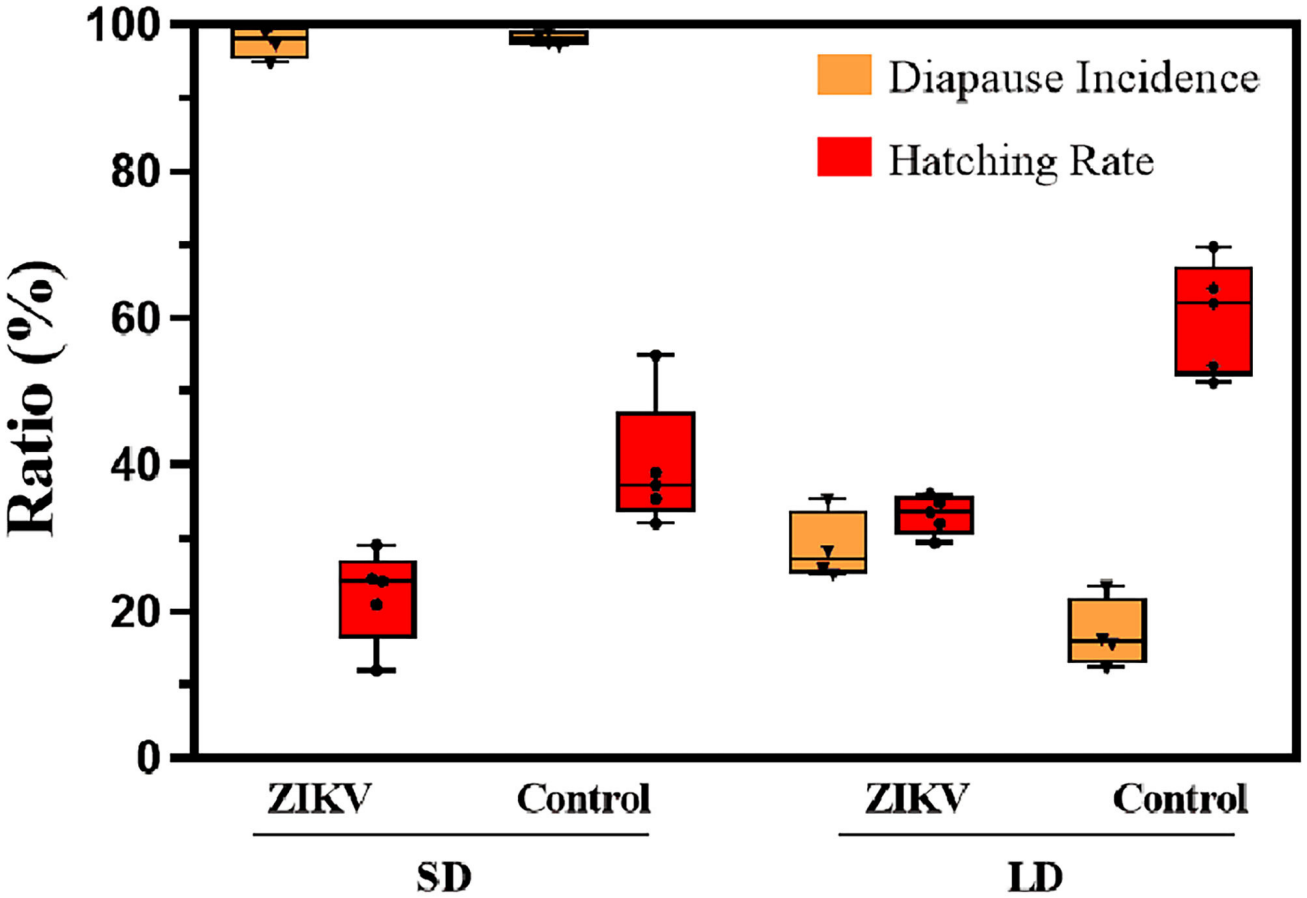
Diapause incidence and hatching rate of ZIKV infection eggs and uninfected control eggs under SD and LD conditions. LD, non-diapause-inducing long-day photoperiod; SD, diapause-inducing short-day photoperiod. Each point represents replicate test. Error bars indicate SDs.

In hatching experiment, all eggs in our simulated winter experiments were aged at least 70 days; therefore, we infer that nearly all eggs will have terminated diapause. After we measured hatching rate in uninfected eggs (Figure 4), 39.7% of diapause eggs were stimulated to terminate diapause from SD photoperiod while over 70% of diapause eggs hatched after 70 days under LD photoperiod conditions (Fisher's exact test, *p* < 0.01). Under SD conditions, 22.1% of the ZIKV infected eggs hatched were lower, which had a significant difference from that of uninfected control group (*p* < 0.05). But the hatching rate in infected eggs from LD group (33.2%) was higher than that in SD group, showing no significant difference (*p* > 0.05).

**Figure 5:**
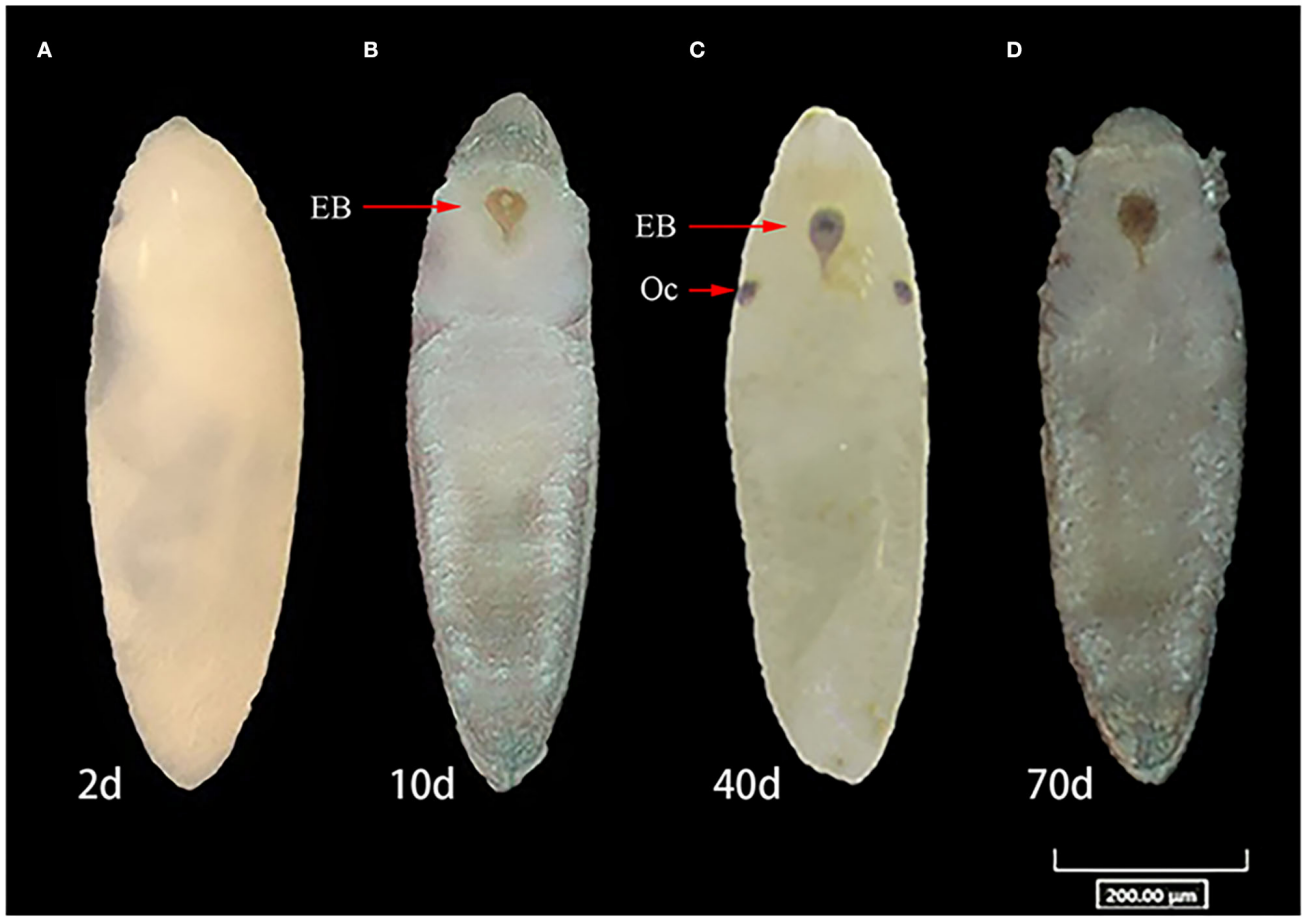
Comparison of developmental stages of diapause, non-diapause eggs of *Ae. albopictus* under short-day photoperiod. **(A)** Egg in embryonic development on 2 days post-oviposition (dpov). **(B)** Ventral view of embryo with appearance of the head with labrum, thorax, abdominal segments, and associated structure on 10 dpov. **(C)** View of diapause embryo with ocelli and egg burster on 40 dpov. **(D)** Diapause eggs on 70 dpov. OC represents ocelli. EB, egg burster.

Diapause incidence was measured in replicates to confirm that SD photoperiod conditions had induced diapause and that LD photoperiod conditions had induced non-diapause. Under SD conditions, the mean diapause incidence across biological replicates was 97.8% in ZIKV-infected eggs, while under LD conditions, the mean diapause incidence was 28.7% of the infected eggs in the hatching experiment. In contrast, the mean diapause incidence of uninfected control eggs in SD and LD groups was 98.1 and 16.9%, respectively, and there was no significant difference compared with infected eggs (Fisher's exact test, *p* > 0.05). Diapause eggs of different developmental stages are shown in Figure 5.

### FIR of Diapause Eggs

We observed the FIR for GC1 with 2/120 of infected larvae in SD group (Table 1). The ZIKV RNA in salivary glands and midguts of offspring was not detected. For LD, detection of ZIKV RNA was not found in filial adults and salivary glands. Only FIR of 1/24 was tested in larvae and midguts.

**Table 1:** Zika virus infection of F1 progeny in the first gonotrophic cycle.

**Photoperiod**	**Tissues**	**FIR (%)**	**RNA copy (log_**10**_)**
SD	Larvae	2/120 (1.67)	3.35 ± 0.24
	salivary glands	0/24 (0)	–
	Midguts	0/24 (0)	–
LD	Larvae	1/120 (0.83)	2.28
	salivary glands	0/24 (0)	–
	Midguts	1/24 (4.17)	2.38

*FIR, filial infection rate; SD, diapause-inducing short-day photoperiod; LD, non-diapause-inducing long-day photoperiod*.

**Figure 6:**
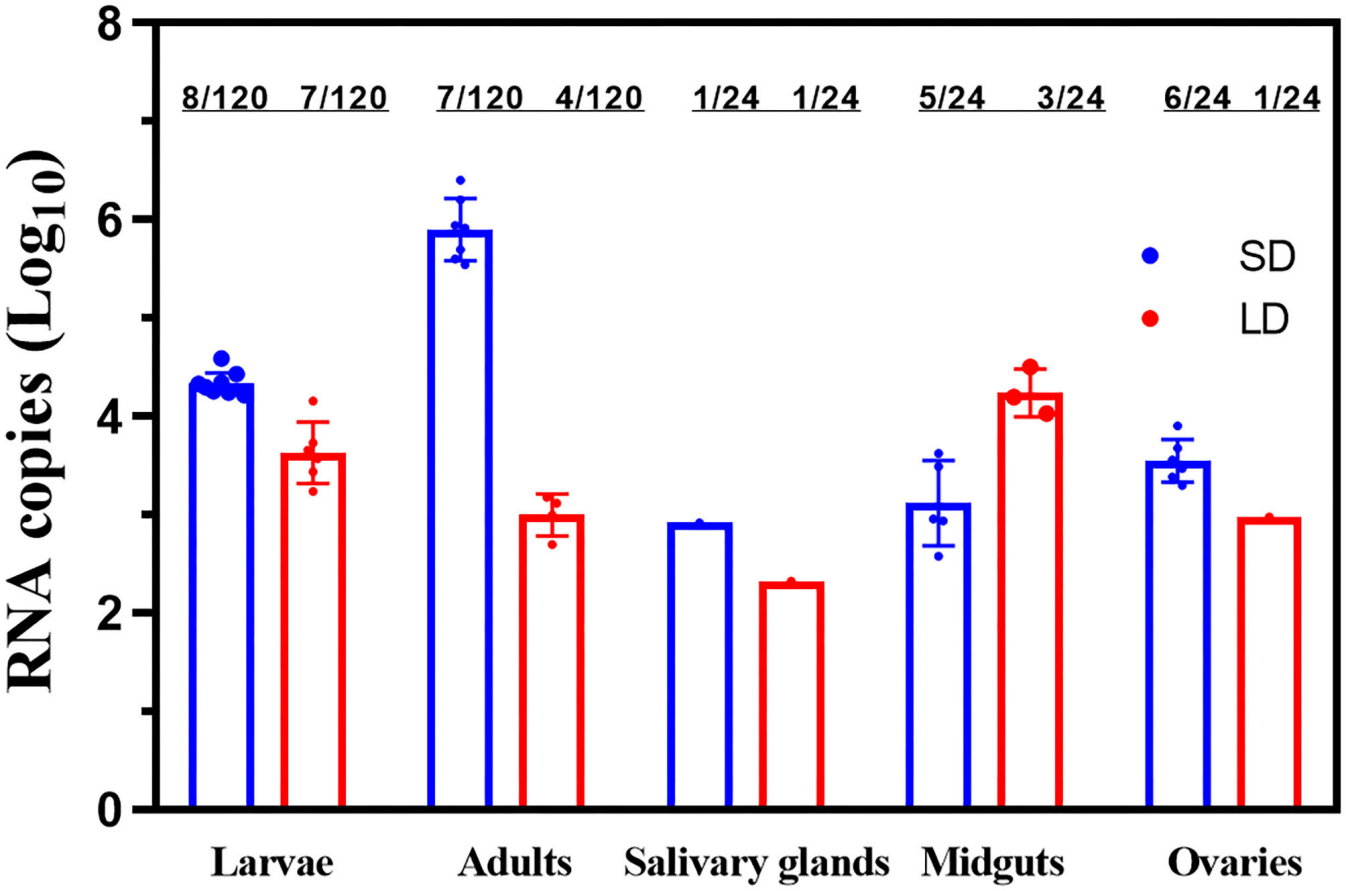
ZIKV RNA copy number in F1 larvae, F1 adults and saliva, salivary glands, midguts, and ovaries of F1 progeny females. LD, a non-diapause-inducing long-day photoperiod; SD, diapause-inducing short-day photoperiod. Each point represents one positive tissue.

But in GC2, the adult females of F1 had an FIR for ZIKV of 7/120 for SD, which decreased to 4/120 in LD group (Figure 6). Both these groups in salivary glands had an FIR of 1/24. FIR for midguts of 5/24 in SD group slightly decreased to 3/24 compared with LD group (Fisher's exact test, *p* > 0.05), while the ovaries of SD and LD had FIRs for F1 of 6/24 and 1/24, respectively. The difference was found to be statistically significant (*p* < 0.05).

Since detection of ZIKV RNA was not found in the offspring adults of GC1, we only carried out biting infant mice experiment with females of GC2. We counted more than one red blotch on the skin of each neonatal mice to make sure all of the neonatal mice were bitten at least once. When neonatal mice were observed for sickness, mice brains were dissected. For SD, 3/10 (30%) of mice brain had viral RNA in SD group, whose RNA copy number was 2.8 ± 0.23 log_10_ RNA copies/μl (Table 2). But 4/10 (40%) of the neonatal mice brain were positive with the RNA copy number of 4.11 ± 1.1 log_10_ RNA copies/μl. The virus titers were 1.7 × 10^2^ and 3 × 10^2^ PFU/ml in LD and SD groups, respectively. The suspensions of F1 progeny adults in GC2 from LD and SD groups were transferred into C6/36 cells after homogenization. The ZIKV infection in C6/36 cells was monitored microscopically for cytopathic effects.

**Table 2:** Detection for ZIKV RNA of the brain after F1 offspring females of the second gonotrophic cycle bit neonatal mice.

**Mosquito**	**NO. of neonatal mice**	**NO. of positive brain**	**Infection rate (%)**	**RNA copy (log_**10**_)**	**Virus titers (PFU/ml)**
SD	10	3	3/10 (30%)	2.81 ± 0.23	1.7 × 10^2^
LD	10	4	4/10 (40%)	4.11 ± 1.1	3 × 10^2^

*SD, diapause-inducing short-day photoperiod; LD, non-diapause-inducing long-day photoperiod*.

## Discussion

Zika virus has quickly spread to many regions across the world. Due to the absence of available vaccines and effective treatment, vector control is the major approach to prevent ZIKV disease (Possas, 2016). Owing to their wide distribution, *Ae. albopictus* mosquitoes might become the primary vector for ZIKV in China. Many studies have confirmed that *Ae. albopictus* mosquitoes could potentially transmit ZIKV (Ciota et al., 2017; Guo et al., 2020). The vector competence of *Ae. albopictus* mosquitoes was strongly related to viral rapid reproduction in the midgut during 0–3 dpi. The viral particles could overcome the midgut barrier and be released into the hemolymph cavity and invade the salivary gland further (Franz et al., 2015). This study showed that ZIKV detection in saliva occurred on 7 dpi. Thus, the results of this study confirmed that *Ae. albopictus* mosquitoes from Beijing could potentially transmit ZIKV.

In *Ae. albopictus*, diapause is under maternal control and responded by producing diapause offspring that overwinter as pharate larvae within the chorion of the egg (Yang, 1988). The adaptive significance of diapause is well understood. Insects in diapause could survive in adverse conditions by increasing nutrient storage and reducing metabolic activity (Hahn and Denlinger, 2011). As described by many studies, *Ae. albopictus* can be easily and consistently induced diapause in the laboratory under SD photoperiods (Poelchau et al., 2014; Huang et al., 2015). Therefore, it is an outstanding model to study the mechanism of photoperiodic diapause, and this mechanism may directly impact disease transmission cycles and the seasonal abundance of vector species (Joy and Sullivan, 2005). In this study, the diapause incidence was 98.1% under diapause-inducing SD photoperiod, and the diapause incidence was 16.9% under LD photoperiod. These results supported earlier observations that diapause incidence ranged from 81.4 to 94.8% under diapause-inducing SD conditions (Huang et al., 2015). Thus, our experimental conditions could consistently induce *Ae. albopictus* diapause in this study. In contrast, hatchability of infected eggs was lower compared to uninfected eggs. The process of viral replication may consume the energy supposed to support development of the eggs (Wang et al., 2018a).

According to research data, ZIKV could survive in diapause eggs until 70 dpov as described. It was worth noting that diapause eggs under SD photoperiod had a relative higher infection rate before 7 dpov and then MIR on 70 dpov would have been minimal, even zero, in GC1, suggesting that the virus infectivity and RNA copy number decreased with time and no virus replication occurred in the eggs finally. These results corroborated the findings of a great deal of previous work (Manuel et al., 2020). Despite the trend of rapid decreasing MIRs, vertical transmission was observed during GC1 (Chaves et al., 2019). This would be a fruitful area for further work to identify within-egg location of virus colonization to demonstrate the existence of vertical transmission.

Compared to GC1, there was a higher level of MIRs in both LD and SD groups in GC2. But the MIRs largely declined from a peak on 3 dpov to a low on 40 dpov. The probable reason could be that the virus may not reach into the inside of the egg through vertical transmission, but the virus is contaminated on the surface of the egg during laying. In the high-temperature condition, the change in MIR in GC2 was not apparent, but RNA copy number of 70 dpov was increasingly higher than those of 40 dpov that indicated that RNA replication occurred in the diapause eggs. Besides, we carried out future studies to assess infection status of offspring mosquito in vertical transmission efficiency. The most obvious finding was that our results reveal ZIKV could be vertically transmitted to their offspring *via* diapause eggs. After diapause eggs hatched, we observed the FIR for GC1 with 2/120 of infected larvae in SD group and 1/120 of infected larvae in LD group. The FIRs of adults' salivary glands were not detected. For GC2, one anticipated finding was that 8/120 larvae and 7/120 offspring adults were tested ZIKV–positive. In accordance with the present results, previous studies have demonstrated that FIR ranged from 1/36 to 1/6,400 (Thangamani et al., 2016). This outcome was contrary to that of Chaves et al., who found that longer incubation time and fewer gonotrophic cycles could help clarify the influence of viral genotype on vertical transmission efficiency and promote vertical transmission (Chaves et al., 2019). In this study, the interesting finding was that viral RNA could exist in larvae, but that viral RNA in adults could not be detected. It was possible that there was transstadial loss of infection after adult mosquitoes emerged (Pereira-Silva et al., 2018). Here, we verified that transstadial transmission was completely efficient.

To our knowledge, this is the first study of detection of ZIKV in diapause eggs of *Ae. albopictus*. Our conclusions show a precise evaluation of vertical transmission, including FIR and the exact viral load of infected progeny. The results highlight that gonotrophic cycle may influence the infection rate of offspring mosquitoes. This study reveals that ZIKV can survive in diapause eggs in winter until the alive virus can rapidly proliferate under appropriate conditions. Thus, these updated findings can be used for preventing ZIKV disease and vector control strategy.

## Data Availability Statement

The original contributions presented in the study are included in the article/supplementary material, further inquiries can be directed to the corresponding author/s.

## Ethics Statement

The animal study was reviewed and approved by Chinese Regulations of Laboratory Animals and the Laboratory Animal Requirements of Environment and Housing Facilities (GB 14925-2010, National Laboratory Animal Standardization Technical Committee).

## Author Contributions

XG and ToZ: conceptualization. QZ, YJ, and ChaL: methodology. TeZ and HZ: investigation. ChuL, DX, and JG: materials and consultation. QZ and YJ: writing – original draft. QZ and XG: writing – review and editing. XG, ToZ, and YD: supervision. All authors contributed to the article and approved the submitted version.

## Funding

This study was supported by grants from the Infective Diseases Prevention and Cure Project of China (No. 2017ZX1030404).

## Conflict of Interest

The authors declare that the research was conducted in the absence of any commercial or financial relationships that could be construed as a potential conflict of interest.

## Publisher's Note

All claims expressed in this article are solely those of the authors and do not necessarily represent those of their affiliated organizations, or those of the publisher, the editors and the reviewers. Any product that may be evaluated in this article, or claim that may be made by its manufacturer, is not guaranteed or endorsed by the publisher.
